# High Genetic Potential for Proteolytic Decomposition in Northern Peatland Ecosystems

**DOI:** 10.1128/AEM.02851-18

**Published:** 2019-05-02

**Authors:** Emily B. Graham, Fan Yang, Sheryl Bell, Kirsten S. Hofmockel

**Affiliations:** a Pacific Northwest National Laboratory, Richland, Washington, USA; b Department of Agricultural & Biosystems Engineering, Iowa State University, Ames, Iowa, USA; c Department of Ecology, Evolution and Organismal Biology, Iowa State University, Ames, Iowa, USA; Wageningen University

**Keywords:** Marcell Experimental Forest, SPRUCE, carbon cycling, metagenomics, microbial community, microbiome, nitrogen cycling, organic nitrogen, soil carbon, soil organic matter

## Abstract

Nitrogen (N) is a scarce nutrient commonly limiting primary productivity. Microbial decomposition of complex carbon (C) into small organic molecules (e.g., free amino acids) has been suggested to supplement biologically fixed N in northern peatlands. We evaluated the microbial (fungal, bacterial, and archaeal) genetic potential for organic N depolymerization in peatlands at Marcell Experimental Forest (MEF) in northern Minnesota. We used guided gene assembly to examine the abundance and diversity of protease genes and further compared them to those of N fixation (*nifH*) genes in shotgun metagenomic data collected across depths and in two distinct peatland environments (bogs and fens). Microbial protease genes greatly outnumbered *nifH* genes, with the most abundant genes (archaeal M1 and bacterial trypsin [S01]) each containing more sequences than all sequences attributed to *nifH*. Bacterial protease gene assemblies were diverse and abundant across depth profiles, indicating a role for bacteria in releasing free amino acids from peptides through depolymerization of older organic material and contrasting with the paradigm of fungal dominance in depolymerization in forest soils. Although protease gene assemblies for fungi were much less abundant overall than those for bacteria, fungi were prevalent in surface samples and therefore may be vital in degrading large soil polymers from fresh plant inputs during the early stage of depolymerization. In total, we demonstrate that depolymerization enzymes from a diverse suite of microorganisms, including understudied bacterial and archaeal lineages, are prevalent within northern peatlands and likely to influence C and N cycling.

**IMPORTANCE** Nitrogen (N) is a common limitation on primary productivity, and its source remains unresolved in northern peatlands that are vulnerable to environmental change. Decomposition of complex organic matter into free amino acids has been proposed as an important N source, but the genetic potential of microorganisms mediating this process has not been examined. Such information can inform possible responses of northern peatlands to environmental change. We show high genetic potential for microbial production of free amino acids across a range of microbial guilds in northern peatlands. In particular, the abundance and diversity of bacterial genes encoding proteolytic activity suggest a predominant role for bacteria in regulating productivity and contrasts with a paradigm of fungal dominance of organic N decomposition. Our results expand our current understanding of coupled carbon and nitrogen cycles in northern peatlands and indicate that understudied bacterial and archaeal lineages may be central in this ecosystem’s response to environmental change.

## Abstract (precis)

Nitrogen (N) is a common limitation on primary productivity, and its source remains unresolved in northern peatlands that are vulnerable to environmental change. Decomposition of complex organic matter into free amino acids has been proposed as an important N source, but the genetic potential of microorganisms mediating this process has not been examined. Such information can inform possible responses of northern peatlands to environmental change. We show high genetic potential for microbial production of free amino acids across a range of microbial guilds in northern peatlands. In particular, the abundance and diversity of bacterial genes encoding proteolytic activity suggest a predominant role for bacteria in regulating productivity and contrasts with a paradigm of fungal dominance of organic N decomposition. Our results expand our current understanding of coupled carbon and nitrogen cycles in northern peatlands and indicate that understudied bacterial and archaeal lineages may be central in this ecosystem’s response to environmental change.

## INTRODUCTION

Understanding the processes that govern coupled carbon (C) and nutrient dynamics in northern peatlands is critical to predicting future biogeochemical cycles. These ecosystems account for 15 to 30% of global soil carbon storage (1–3), occurring primarily within layers of partially decomposed plant materials where the nitrogen (N) content is low (4, 5). Nitrogen is a critical nutrient regulating primary productivity in many terrestrial ecosystems (6) and can dictate below-ground carbon storage through impacts on soil organic matter decomposition (7, 8). Ombrotrophic peatlands are characterized by *Sphagnum* moss that has a comparatively large N requirement (approximately 40 to 50 kg ha^−1^ year^−1^ of N [9–12]). Nitrogen fixation historically has been considered to be the primary N source in peatlands (13–19). However, previous work has shown that N fixation alone cannot meet peatland N requirements (5, 20), and many studies have demonstrated the importance of organic molecules in fulfilling N demand (21–26). Symbiotic fungi have been associated with organic N acquisition (23, 26), but there is an increasing appreciation for the role of bacteria in this process. Despite these advances, our understanding of the genetic mechanisms mediating N availability remains nascent. We address this knowledge gap by exploring the genetic potential of peatland microbiomes to decompose polymeric organic N and subsequently influence peatland C and N cycles.

Depolymerization of proteinaceous organic material is an important pathway for generating bioavailable N in wide range of systems, including boreal forests, and is often considered a fungal trait (5, 7, 21, 23, 27–29). Depolymerization decomposes polymeric organic material into monomers and amino acids that can be used as C and N sources by soil microorganisms and plants (20, 21, 23, 30, 31). Several studies from terrestrial ecosystems under strong inorganic N limitation have shown that organic N, and free amino acids in particular, can be used directly by plants (20, 21, 23, 30, 31). Additionally, microorganisms (defined here as bacteria, archaea, and fungi) secrete extracellular proteases into soils to carry out organic matter depolymerization. Proteases are highly diverse and ubiquitous in soil and provide a large proportion of bioavailable N (32, 33). These enzymes catalyze the initial hydrolysis of proteins into smaller organic molecules such as oligopeptides and amino acids that can be subsequently acquired by plants (32).

In peatlands, fungi are considered more important than bacteria or archaea in proteolytic activity and decomposition more generally, particularly within the surface layer (34–36). Symbiotic ectomycorrhizal and ericoid fungi (EEM), which are supplied with C by a host plant, are especially relevant to organic N depolymerization in peatlands through N mining (37–39). EEM have been suggested to acquire N from soil organic matter (20, 40–42) and enable plants to directly compete with free-living microorganisms for N (43–45) to such an extent that Orwin et al. (46) posited a critical role for EEM in generating microbial N limitation of decomposition by enhancing plant N uptake. This fungally mediated plant organic N uptake may be particularly important in N-poor boreal ecosystems (20, 21). In these systems, free-living microorganisms should retain amino acids for growth instead of mineralizing organic N (23, 47). However, empirical evidence supporting the notion that fungi dominate proteolytic activity is sparse and derived primarily from correlative studies.

Little is known about the roles that various other microorganisms may play in peatland organic N depolymerization, and the genes that encode microbial proteases may provide valuable insight into the coupling of C and N cycles in these systems. Recent work has suggested substantial involvement of bacteria in protein depolymerization. For example, Lin et al. (35, 48) indicated that bacteria may outcompete fungal communities for plant-derived substrates, including large polymeric molecules. Consistent with this work, Bragina et al. (49) demonstrated that peatland *Sphagnum* moss microbiomes contain a high abundance of genes involved in N cycling and recalcitrant organic matter decomposition. The involvement of archaeal proteases in peatland organic N decomposition remains largely unexplored.

Here, we evaluated microbial proteolytic potential relative to N fixation within the Marcell Experimental Forest (MEF) by examining the genes encoding a suite of microbial proteases versus the *nifH* gene, with is well attributed to N fixation. Because the peatlands are highly acidic (pH 3 to 4) and organic polymers have bulky structures, we focused on microbial genes encoding extracellular proteases previously identified as active in acidic environments (50–52). This led to the identification of 19 extracellular protease gene families according to the MEROPS database (53). We also included one intracellular protease gene (encoding aminopeptidase, M1 family) in the study due to the encoded protein’s role in producing single amino acids during depolymerization (50–52). We evaluated the distributions of these protease genes, as well as three housekeeping genes and the *nifH* gene, in six metagenomes across two peatland environments and three depths. Though our statistical power was limited, we know of no other work investigating the genetic potential of peatlands for organic N degradation across microbial domains, and our results contrast with the existing paradigm of fungal dominance in proteolytic decomposition from upland ecosystems. We leverage work demonstrating differences in organic matter cycling between hydrologically defined environments within peatlands (e.g., bogs versus fens) as a basis for potential differences in proteolytic activity across environments (35, 54–56). Previous research has also shown declines in fungal biomass depth as oxygen and root exudates become depleted in peatland, so we examined differential vertical stratification patterns in proteases between microbial domains (34, 57, 58). Our objectives were to provide a foundation for characterizing the mechanisms driving N availability and C decomposition patterns in peatlands through investigating shifts in proteolytic potential across (i) fungal, bacterial, and archaeal domains, (ii) peatland environment types, and (iii) depth profiles.

## RESULTS AND DISCUSSION

Nitrogen fixation has long been considered the primary N source for peatlands (13–18), but N fixation alone cannot meet ecosystem N requirements (5, 20). Similarly, N assimilation has been shown to exceed gross mineralization in northern ecosystems (20, 21), and intact amino acid assimilation has been recognized as a potentially important source to meet N demand (23, 31, 37, 59). Previous work suggested that microbial proteases may be a missing link in northern peatland C and N cycling (31, 60). We investigated the proteolytic potential of peatland microbiomes across depth and environment type. Our work contrasts with the paradigm of fungal dominance in depolymerization processes and suggests that niche complementarity among diverse microorganisms is likely to play a substantial role in C and N cycling within northern peatlands.

Out of 24 genes investigated, we successfully assembled sequences aligned to 13 genes (see Tables S1 and S2 in the supplemental material). The assembled groups include three housekeeping genes (*rplB*, *rpb2*_4, and *rpb2*_7), nine protease genes (eight of which encode extracellular proteases), and the *nifH* gene. Approximately 34% of the fully covered contigs were annotated as housekeeping genes, 5% were annotated as *nifH* genes, and 61% were annotated as protease genes. Among all metagenomic reads mapped to the annotated fully covered contigs, approximately 83.5% were bacterial, 16% were archaeal, and 0.5% were fungal (Table 1).

**TABLE 1:** Distribution of reads mapped to contigs identified as housekeeping genes, N fixation genes, and protease genes and the distributions of all mapped reads among archaea, bacteria, and fungi

Sample	Peatland depth (cm)	No. of reads					
		Housekeeping genes	N fixation genes	Protease genes	Total		
					Archaea	Bacteria	Fungi
Fen1_−10	Fen −10	3,822	559	10,296	1,851	12,754	72
Fen2_−10	Fen −10	2,506	469	5,757	894	7,701	137
T3M_−10	Bog −10	2,680	339	7,704	1,418	9,201	104
T3F_−10	Bog −10	2,785	736	6,194	663	9,003	49
T3F_−50	Bog −50	8,003	1,061	22,256	4,733	26,538	49
T3F_−100	Bog −100	4,174	266	11,420	4,933	10,888	39

**FIG 1:**
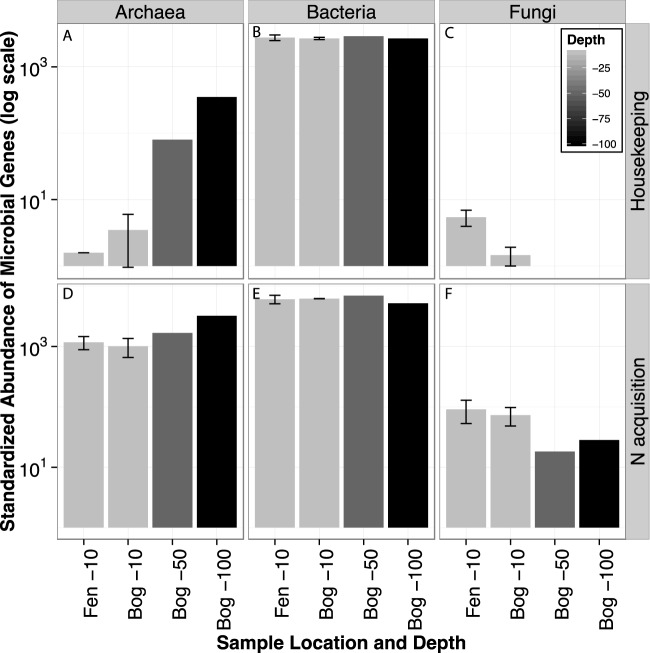
Standardized abundance of identified microbial genes in MEF peatlands through sampling depth. (A to C) Distribution of housekeeping genes. (D to F) Distribution of N acquisition genes. For location-depth combinations with two samples (Fen −10 and Bog −10), the bar height represents the average standardized gene abundance of two samples and the hatched lines represent the maximum and minimum values.

**FIG 2:**
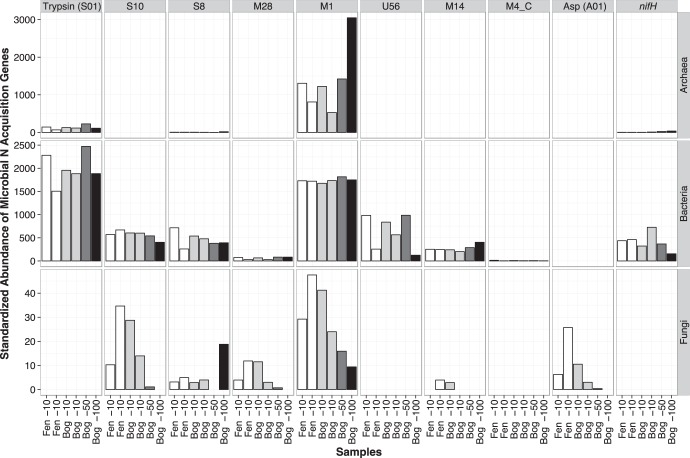
Abundance of identified nitrogen acquisition gene assemblies and their taxonomic distribution. Microbial proteases greatly outnumbered *nifH* genes (last column), with the most abundant genes (bacterial trypsin [S01] [column 1] and archaeal M1 [column 4]) each containing more sequences than all sequences attributed to *nifH*. Additionally, the relative abundance of most bacterial protease genes did not differ across depth profiles. Samples from the same depth and environment are colored identically, as denoted on the *x* axis. The sampling depth increases from left to right. A description of samples is given in Table 1.

We reveal unique niches for fungal, bacterial, and archaeal proteolytic potential, as housekeeping and protease genes from each kingdom showed distinct stratification patterns across depth. Within housekeeping genes, the standardized abundance of bacterial genes was similar across depth profiles, whereas the standardized fungal gene abundance decreased and archaeal gene abundance increased along the sampling depth (Fig. 1). Protease-encoding genes differed among archaea, bacteria, and fungi (Fig. 2). Among nine protease genes, eight were identified in bacteria, six were identified in fungi, and only three were identified in archaea. With some exceptions, archaeal protease genes increased with sampling depth, fungal protease genes decreased with depth, and bacterial protease genes varied throughout depth profiles.

Fungi were mostly found in the acrotelm, which constitutes the peat surface and is more oxygenated than deeper peat layers (Fig. 2). The acrotelm contains higher concentrations of C inputs from newly derived plant material, such as lignin-, cellulose-, and protein-based polymers (61). Lignin in particular requires oxygen for decomposition due to its comparatively high chemical complexity, such that some bonds cannot be readily cleaved by hydrolases or reductases, and other work has indicated an association between fungal proteases and lignin decomposition under N-limited conditions (62, 63). Previous work in this system has also shown that carbohydrates are enriched in the surface layer, while amino sugars and saccharides increase with depth (35). We therefore suggest that fungi are particularly relevant players in early stages of decomposition, in which fresh plant material is degraded into small oligopeptides, consistent with other evidence showing that fungi are unlikely to metabolize complex organic molecules found at depth in peatlands (58, 64).

Bacteria were the most abundant sequences detected regardless of depth or environment, in line with previous work by Lin et al. (55), and the presence of large numbers of bacterial proteases relative to those of fungi and archaea signifies a possible dominance of bacterial depolymerization in northern peatlands. Bacterial housekeeping genes and protease genes were approximately equally abundant throughout depth, and their sheer abundance suggests that at the community scale, bacteria may outcompete fungi and archaea in proteolytic decomposition. Additionally, bacterial diversity throughout the depth profile, despite relatively constant abundance, indicates plasticity in bacterial resource use across a variety of organic matter degradation states (Fig. 2).

Complementary to fungal and bacterial niche space, archaea had clear advantage in deep peat. Archaeal protease genes were more abundant in the mesotelm (25 to 50 cm) and catotelm (>50 cm) than in the acrotelm (0 to 10 cm). Lin et al. (55) noted the presence of archaea more generally at depth in peat, reaching up to 60% of total small-subunit rRNA gene sequences below 75 cm. Archaea are found in a variety of anaerobic and extreme environments, and specifically, methanogenic archaea are tolerant of low-oxygen conditions that persist in deep peat. Indeed, many of the protease genes we observed in deep peat were associated with methanogenic lineages (see Fig. S1 in the supplemental material). The presence of archaeal proteases at depth suggests that archaea may be vital to the decomposition of the oldest and most humified organic materials stored within peatlands. In total, the consistent differences in abundance and diversity of fungi, bacteria, and archaea across the three peat depths of the bog and fen suggest that niche partitioning across redox profiles may substantially influence the mechanisms of microbial decomposition.

With respect to differences in microbiomes across environments, we found few differences in standardized gene abundance between bog (*n* = 3) and fen (*n* = 2) acrotelm samples, except that fungal genes were more abundant in fen acrotelm than in bog acrotelm samples (Fig. 1). However, three proteases (M14, M4_C, and asp [family A1]) were at least 12% more abundant in samples from the fen acrotelm than in those from bog acrotelm, and assemblies resembling U56 were less abundant in the fen than in the bog (Fig. 2). Remaining protease genes were less than 10% different across environments (Table 2).

**TABLE 2:**
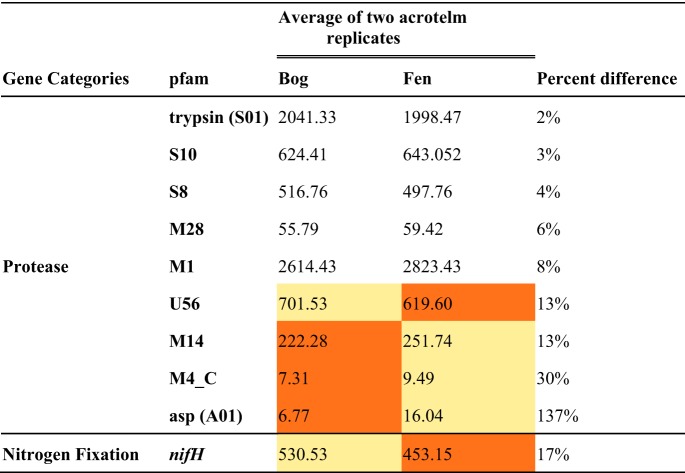
Standardized abundance of N acquisition genes in acrotelm peat samples^*a*^

a The values represent the average for two replicates of acrotelm samples from each geological location. The lighter color indicates a higher value. The percent difference was calculated as 100 × (*H*/*L* − 1), where *H* is the high value and *L* is the low value.

Regardless of environment type or depth, bacterial protease abundance and diversity as a whole indicate a wide variety of possible niches for C- and N-cycling bacteria within peatlands. Aminopeptidase N (M1), which cleaves peptides and produces N-terminal amino acid residues (65, 66), was the most prevalent microbial protease. Work in other systems has shown that bacterial aminopeptidase N proteases can account for 99% of alanine released from substrate hydrolysis (67) and that they are critical in generating bioavailable organic N via microbial biomass turnover (68). Thus, we highlight the M1 gene family as encoding key enzymes for understanding peatland N cycles.

**FIG 3:**
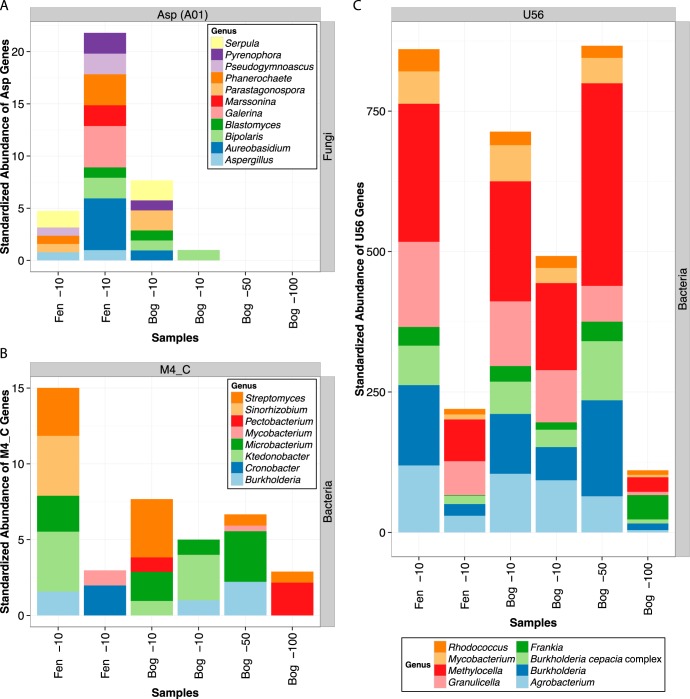
Phylogenetic distribution of the most abundant fungal asp (A01) (A), bacterial M4_C (B), and bacterial U56 (C) genes with variation across environments. Genus-level data are presented.

Beyond the protease M1 family, extracellular protease genes were highly diverse, and we propose that specific extracellular proteases we identified may fill unique steps in decomposition of plant material (Fig. 3) (69–71). In particular, asp (A01) genes were uniquely detected in fungi, while M4_C and U56 genes were found only in bacteria (Fig. 2). Each of these proteases contained a wide variety of genera with differences across both depth and environment type, and the abundance of asp (A01) genes in the acrotelm appeared to be inversely related to that of bacterial M4_C and U56 genes (Fig. 3). Previous work has shown that the fens at Marcell Experimental Forest have roughly 10% more dissolved organic C than bogs and that this difference in geochemistry explains most of the variation in microbiome composition between fen and bog samples (48). We note that fungal protease genes in particular are more abundant in the fen acrotelm than in the bog acrotelm (the layer in which most fungal biomass was found), supporting a role for fungal proteases in the decomposition of fresh carbon-rich plant material. asp (A01) genes are commonly associated with fungal wood decomposition (62, 63). asp (A01) proteases may therefore play an important role in the early stage of peatland depolymerization, in which large polymeric molecules are degraded. asp (A01) genes consistent with the genera *Phanerochaete*, *Pseudogymnoascus*, and *Aspergillus* were detected in fen samples only (Fig. 3A), and their presence in the more C-rich fen environment may denote a distinct ecological niche for these organisms in proteolytic decomposition within fen-dominated peatlands.

Bacterial M4_C genes were particularly abundant in one fen acrotelm sample (Fen1_−10) and were taxonomically diverse within acrotelm samples more generally (Fig. 3B). M4_C genes are involved in the hydrolysis of proteins with large hydrophobic groups at P1′ (Table S2). Although bacterial and archaeal protease gene abundances were mostly similar across environments, protease genes classified as U56 were 12% more abundant in the bog than in the fen and had diversity similar to that of the M4_C gene, with *Methylocella* and *Burkholderia* as the most abundant genera (Table 2 and Fig. 3C). We therefore propose that U56 (formerly linocin M18 [72]) may be an essential protease in peatlands. U56 is thought to hydrolyze chymotrypsin and trypsin into its component amino acids (Table S2). Though we did not explore the niches of these organisms beyond proteolytic activity, *Methylocella* spp. are commonly associated with methanotrophy (73), and *Burkholderia* spp. are functionally diverse but often are considered to be plant-associated nitrogen fixers (74, 75). The abundance of proteases associated with *Methylocella* and *Burkholderia* merits future investigation into their role in peatland biogeochemistry.

While we support previous work showing a role for N fixation in peatlands, particularly in surface peat (35), we suggest that microbial depolymerization complements N fixation in highly N-limited ecosystems. Our work is consistent with the recently proposed “LAH N acquisition strategy” framework, in which organic N decomposition supplements N fixation (76). The concept uses a cost-benefit framework to propose that proteolytic activity can be a preferred N acquisition strategy for microorganisms due to the high enzymatic cost of N fixation. We were able to assemble >21,000 archaeal and bacterial *nifH* genes, but *nifH* genes were less prevalent than the most abundant protease genes in each microbial domain (archaeal M1 and bacterial trypsin [S1 family]) (Fig. 2), in line with the observation that N accumulation in boreal ecosystems exceeded N deposition from atmosphere by 12- to 25-fold (10). *nifH* genes were detected in both archaea and bacteria, and approximately 97% of the *nifH* genes detected were bacterial. Nitrogen fixation is not known to be mediated by fungal communities (77). *Sphagnum* is known to harbor a diversity of N-fixing symbionts (49), including *Cyanobacteria* observed here (78), and is likely vital to atmospheric N fixation in peatlands.

### Conclusion.

We explored the genetic potential for organic matter depolymerization in a northern peatland based on previous work indicating that microbial proteases may be critical in understanding in C and N availability within these ecosystems (31, 60). We found that fungal protease genes were abundant in the acrotelm (surface layer), but bacterial proteolytic potential was orders of magnitude greater and distributed through depth profiles. Bacterial protease gene abundance was consistent across environments, and fungal protease genes were more prevalent in the higher-C fen environment. In contrast to the case for fungi and bacteria, the prevalence of archaeal protease genes at depth suggests an importance of these organisms in C and N availability below the rooting zone in peatlands. We also showed a diversity of protease genes that suggests strong niche complementarity among microorganisms with different physiologies. We identify proteases M1, U56, and asp (A01) as proteases that may be particularly important within northern peatlands. In total, protease genes greatly outnumbered *nifH* genes attributed to N fixation, emphasizing their role in peatland C and N cycles. We contrast the paradigm of fungal dominance in depolymerization processes and suggest that bacteria are imperative in releasing free amino acids from peptides through depolymerization of older organic material. Our work demonstrates a high genetic potential for depolymerization from a diverse suite of microorganisms beyond those typically considered, and we urge a broader perspective on the organisms mediating C and N cycles in northern peatlands.

## MATERIALS AND METHODS

### Sample description.

A large-scale field manipulation experiment known as Spruce and Peatland Responses Under Changing Environments (SPRUCE) was initiated at the Marcell Experimental Forest (MEF), MN, USA, by the U.S. Department of Energy, U.S. Department of Agriculture (USDA) Forest Service, and Oak Ridge National Laboratory (http://mnspruce.ornl.gov/). The MEF itself is a 8.1-hectare acidic, forested bog (47°30′31.132′′N, 93°27′15.146′′W). Sites within the MEF are classified based on their trophic status and water source as ombrotrophic bogs (receiving precipitation only) or minerotrophic fens (fed by both groundwater and precipitation [48, 79]). Although fens are frequently considered more nutrient rich than bogs, both types of peatlands are highly limited in inorganic N. A full characterization of the field site, including peatland hydrology and vegetation, is described by Sebestyen et al. (80). Further information on samples is available from Lin et al. (35, 55).

Six metagenomic libraries were obtained from MEF in February 2012 as described by Lin et al. (35). Briefly, peat cores were collected from hollows in bogs and fens and sectioned from 0 to 10 cm (acrotelm), 25 to 50 cm (mesotelm), and 75 to 100 cm (catotelm). While the water table was near the surface of the *Sphagnum* layer at the time of sampling, MEF experiences large seasonal variation in water table height, with the acrotelm and mesotelm layers frequently exposed to oxygen (81). For example, in the year of sampling, the water table depth fell to −20 cm below the *Sphagnum* layer (81), and microbiomes in the acrotelm contained many aerobic microorganisms (55). Each core section was homogenized. Two acrotelm samples (0 to −10 cm) were collected from bog lake fen (i.e., samples Fen1_−10 and Fen2_−10), two acrotelm samples (0 to −10 cm) from SPRUCE bog (i.e., samples T3M_−10 and T3F_−10), one mesotelm sample (−25 to −50 cm) from SPRUCE bog (i.e., sample T3F_−50), and one catotelm sample (−75 to −100 cm) from SPRUCE bog (i.e., sample T3F_−100). Sample collection is extremely limited at MEF to preserve the integrity of the SPRUCE experiment. As such, our sample numbers are not conducive to statistical analysis, but the metabolic insight generated by our in-depth metagenomic analysis merits deeper investigation into the respective roles of microbial domains in coupled C-N cycles in northern peatlands.

As described by Lin et al. (35, 55), samples differed in physicochemical properties across the depth layers. Sequencing coverage for each metagenome increased with depth and ranged from 42% to 86% (Table 1). Metagenomes were generally consistent between samples from the same depth and site, suggesting that the results reported here are likely to be robust (further details were provided by Lin et al. [35]). DNA-based approaches reflect microbial potential to catalyze a given process rather than gross activity rates; however, metagenomic sequences are often used as a proxy for identifying biogeochemical mechanisms of interest. The phylogenetic distribution of microorganisms in each sample at the community level was presented by Lin et al. (55), and that at the protease level is shown in Fig. S2 in the supplemental material. Microorganisms belonging to *Acidobacteria*, *Proteobacteria*, *Actinobacteria*, and *Verrucomicrobia* were common at both sites, with methanogenic archaea present at depth. Whole-genome shotgun metagenome sequences are available in MG-RAST (35, 55).

### HMM construction.

We used 24 hidden Markov models (HMMs) constructed based on protein sequences (82) to investigate the microbial genetic potential in N acquisition in MEF peatlands, 20 of which were for microbial protease genes, one for the nitrogenase gene (*nifH*), and three for microbial single-copy housekeeping genes (see Table S2 in the supplemental material). Nitrogenase enzymes are encoded by three genes (*nifH*, *nifD*, and *nifK* [reviewed in reference 83]); however *nifH* is the most commonly used marker gene for nitrogenase potential (77, 84). Evidence suggests that *nifD*- and *nifK*-based assays are consistent with *nifH*-based results (85). We use “N acquisition genes” and related terms to represent all protease genes and *nifH*. The abundance of N acquisition genes is therefore the sum of the abundance of all protease genes plus the abundance of *nifH*. The housekeeping genes used as bacterial, fungal, and archaeal markers are genes encoding ribosomal protein L2 (*rplB*), RNA polymerase second-largest subunit domain 4 (RPB2_4), and domain 7 (RPB2_7), respectively. We use the term “housekeeping genes” to represent these domain-specific single-copy housekeeping genes. The abundance of housekeeping genes is calculated as the sum of the abundance all housekeeping genes. The HMMs for *nifH* and *rplB* genes are available in Ribosomal Database Project (RDP) Fungene repository (86). Models for RPB2_4 and RPB2_7 were obtained from Pfam database (http://pfam.xfam.org). The protease genes (one intracellular and 19 extracellular) were selected based on literature characterizations (50–52). Though there are hundreds of protease genes in the MEROPS database (53), most encode intracellular enzymes not thought to be involved in decomposition or unsuitable for the acidic environments. We therefore targeted protease genes both known to encode functions relevant to peatland decomposition and known to be expressed and active in previous lab assays (50–52). To construct the most informative HMM models for targeted genes, well-studied gene sequences were first selected from existing literature. These seed sequences were cross-checked against the reviewed protein database Swiss-Prot (http://www.uniprot.org/). Genes encoding proteases were also searched against the MEROPS peptidase database (http://merops.sanger.ac.uk/) to confirm their protease identity. Protein families and existing protein HMM models were queried from Pfam database. The retrieved Pfam HMMs were then used to extrapolate archaeal, bacterial, and fungal reference protein sequences from the UniProt database. Pfam HMMs were used to search against Swiss-Prot at different cutoffs (E value or Bit score) to ensure model accuracy. If existing Pfam HMMs could not accurately query sequences, a set of well-annotated sequences would be used to construct new models (Table S2). Reference protein sequences were retrieved from UniProt and aligned using finalized HMMs.

### Guided metagenomic assembly.

All metagenomic reads were filtered by using RDP SeqFilters (87) to a minimal average read quality of *Q* = 25. Genes with HMM models (Table S2) were assembled from combined filtered reads by using a modified RDP Xander skeleton analysis pipeline (https://github.com/fishjord/xander_analysis_skel). Briefly, a De Bruijn graph was built for the combined shotgun metagenome data set. Potential gene start points (*K*-mer starts, *k* = 30 nucleotides) were identified from each gene reference sequence. Local assembling was carried out by searching constructed De Bruijn graphs at the given gene start points. These local assemblages were then merged to form the longest contigs possible. The final merged nucleotide sequences were dereplicated using CD-Hit 4.6.1 (-c 1.0) (88) to identify the longest unique contigs.

### Data processing.

All quality filtered reads were mapped (Bowtie2.2.5) (89) against the dereplicated merged contigs. Only contigs that were 100% covered (median base coverage = 1) were considered. The biological information of these fully covered contigs was identified using Basic Alignment Searching Tool (blastx) 2.2.30+; the best-matching sequences with E values of ≤1 × 10^−5^ were kept (90). UniProt (UniProtKB release 10, 2014) was used as the annotation database.

The final gene abundance per peat sample was determined by mapping reads from each metagenome to fully covered contigs. The mapping results indicated that ∼9% of reads were mapped onto the contigs once only and the majority of the fungal housekeeping contigs were mapped once. Hence, for downstream analyses, we included all reads mapped to final fully covered contigs at least once (see Table S3 in the supplemental material). The mapped read abundances were standardized by sequencing depth for comparisons among samples. Gene abundance was used to infer the abundance of mapped reads to fully covered contigs. Final data analyses and visualization were done in R 3.1.0 (91) with packages plyr (92) and ggplot2 (93).

## Supplementary Material

Supplemental file 1
