# Supplementary material for: High Genetic Potential for Proteolytic Decomposition in Northern Peatland Ecosystems
Source: Appl Environ Microbiol. 2019 May 2;85(10):e02851-18. doi: 10.1128/AEM.02851-18 (PMC6498154; doi:10.1128/AEM.02851-18)
Supplement: Supplemental file 1 [file AEM.02851-18-s0001.pdf]

Table S1. The 13 genes successfully assembled.

	Protein Family (pfam)	Number of Gene Starts	Number of Merged Contigs	Unique Contigs	Fully covered contigs
House-Keeping Genes	**rplb**	140627	131882	18779	2729
	**rpb2_7**	1089	722	91	62
	**rpb2_4**	221	74	8	6
N Fixing Genes	**nifh**	39592	29298	3010	410
Protease Genes	**Asp (family A1)**	2277	390	55	52
	**M1**	77454	39343	6673	1608
	**M14**	15955	2170	417	243
	**M28**	36002	552	65	53
	**M4_C**	760	185	29	26
	**S10**	40281	4124	762	459
	**S8**	61669	6758	1301	950
	**Trypsin (S01)**	140032	18952	3958	950
	**U56**	17473	11636	1845	599

Table S2. Gene groups used in guided gene assembling. The second column shows the minimal e-valule used in querying reference sequences. Column HMM Model indicates whether the model was readily available through Pfam or contumely built. The last column shows the seed sequences used in gene identification.

Gene Categories	Protein Family (pfam)	Reference Proten Sequence Min Evalue	HMM Model	Seed Uniprot Sequence ID (position)	Functions
Protease	M1 (EC:3.4.11.2)	1.00E-05	Pfam	Q48656 (2-849)	intracellular, release small amino acids from peptides
	Amidase	1.00E-05	Staphylococcus aureus, lytA gene searched agained Swissprot and selected lysostaphin, glycyl-glycine endopeptidase, N-acetylmuramoyl-L-alanine amidase, and endolysin	P24556, Q38653, P39800, P14892, O34391, P54450, P24808, O05156, P10547, P10548,	cell-wall turnover
	Asp (family A1)	7.60E-01	Pfam	Q00663 (61-394)	fungal acid protease preferentially cleaves the C-terminal of hydrophobic amino acids
	Beta-lytic metallopeptidase (EC 3.4.24.32)	1E-50 (1E-75 gives the same results)	Beta-lytic metalloendopeptidase from Achromobacter lyticus was searched against Swissprot. Beta-lytic metalloendopepetidase form Achromobacter lytiucs and Lysobacter enzymogenes were selected to be seeds	P27458, P00801	cleave lysyl peptide bond (EC 3.4.24.32)
	C11	1.00E-05	Pfam	P09870 (1-526)	nutrient acquisition by cleaving arginine peptide bond
	C47	1.00E-02	Pfam	Q5HKF6 (222-395)	cell virulence, cleaves hormone peptides
	Lysyl endopeptidase (EC 3.4.21.50)	1.00E-75	Achromobacter lyricus, Protease 1, searched agaist Swissprot. Lysyl endorpeptidase and protease 1 were selected	P15636, Q7M135, Q9HWK6	cleave lysyl peptide bond (EC 3.4.21.50).
	M10	1.00E-05	Pfam	P16316 (16-481)	preferentially cleave hydrophophic residues (EC: 3.4.24.40).
	M14	3.00E-08	Pfam	D4B5N0 (114-416)	pathogenicity
	M23_staph	1.00E-153	Pfam	P10547 (248-493)	lyse cells by hydrolyzing the polyglycine interpetide bridges of the peptidoglycan
	M26_N	1.00E-05	Pfam	Q97QP7 (100-2004)	cell virulence, cleaves hormone peptides
	M28	1.00E-05	Built based 55 fungal M28 extracellular protease protein sequences (HMMER3.0)	P81715 (38-438)	Degrades leupeptin into three components, acetyl-leucine, leucine and argininal
	M35	1.00E-02	Pfam	P00733 (43-255)	catalyzes carboxypeptidation and transpeptidation reactions
	M4_C	1.00E-05	Pfam	P14756 (198-498)	Hydrolysis of proteins with bulky hydrophobic group at P1'
	S10	1.00E-05	Pfam	P00729 (112-532)	degradation of small peptides
	S37_S28	1.00E-05	Built based on Uniprot sequences found according to pfam S37 HMM model with 1E-39 cutoff (HMMER3.0)	P83615 (34-477)	proline-specific tripeptidyl aminopeptidase and tetrapeptidyl aminopeptidase
	S8	1e-0.0098	Pfam	P00782 (108-382)	digest casein for lactic acid bacteria
	staphylokinase	1.00E-05	Pfam	P10520 (27-440)	not a protease, but it activates plasminogen by complexing with it
	Trypsin (Family S1)	1.00E-05	Pfam	P00778 (200-397)	Preferential cleaves Ala and Val residues in bacterial cell walls, elastin and other proteins.
	U56	1.00E-05	Swissprot U56 family sequences (B1L7S2, A5IJ00, Q9WZP2, Q45296)	Q45296	homomultimeric peptidase hydrolyses substrates of chymotrypsin and trypsin
Nitrogen Fixation	nifH	bit score 70	Fungene	Readily available	N2 fixation
House Keeping	rplB	1.00E-05	Fungene	Readily available	Bacteria marker
	rpb2_7	5.00E-03	Pfam	Readily available, pfam seed	Archaea marker
	rpb2_4	5.00E-03	Pfam	Readily available, pfam seed	Fungi marker

Table S3. The abundance of raw reads, quality filtered reads, unique contigs assembled, and assembled genes that are similar to genes in UniProt database.

Samples	Raw Reads	Q≥25	Unique Contigs Assembled	Fully Covered Unique Contigs[a]	# of Reads Mapped[b] (min 2)	# of Reads Mapped[b] (min 1)
**Fen -10**	5,932,150,968	39,366,506	-	-	13,176	14,677
**Fen -10**	4,484,850,217	31,389,057	-	-	7,453	8,732
**Bog -10**	4,828,848,088	32,433,476	-	-	9,074	10,723
**Bog -10**	4,610,093,913	31,100,079	-	-	8,134	9,715
**Bog -50**	10,217,289,715	83,935,703	-	-	30,340	31,320
**Bog -100**	5,346,383,014	43,018,379	-	-	15,121	15,860
**Total**	35,419,615,915	261,243,200	36,993	8,147		

a. Unique contigs that can be completely covered by reads (# of read bases/# of contig bases ≥ 1)

b. The number of quality filtered reads that can be mapped onto the fully covered unique contigs with a minimum presence of 2 (min 2), or 1 (min 1).

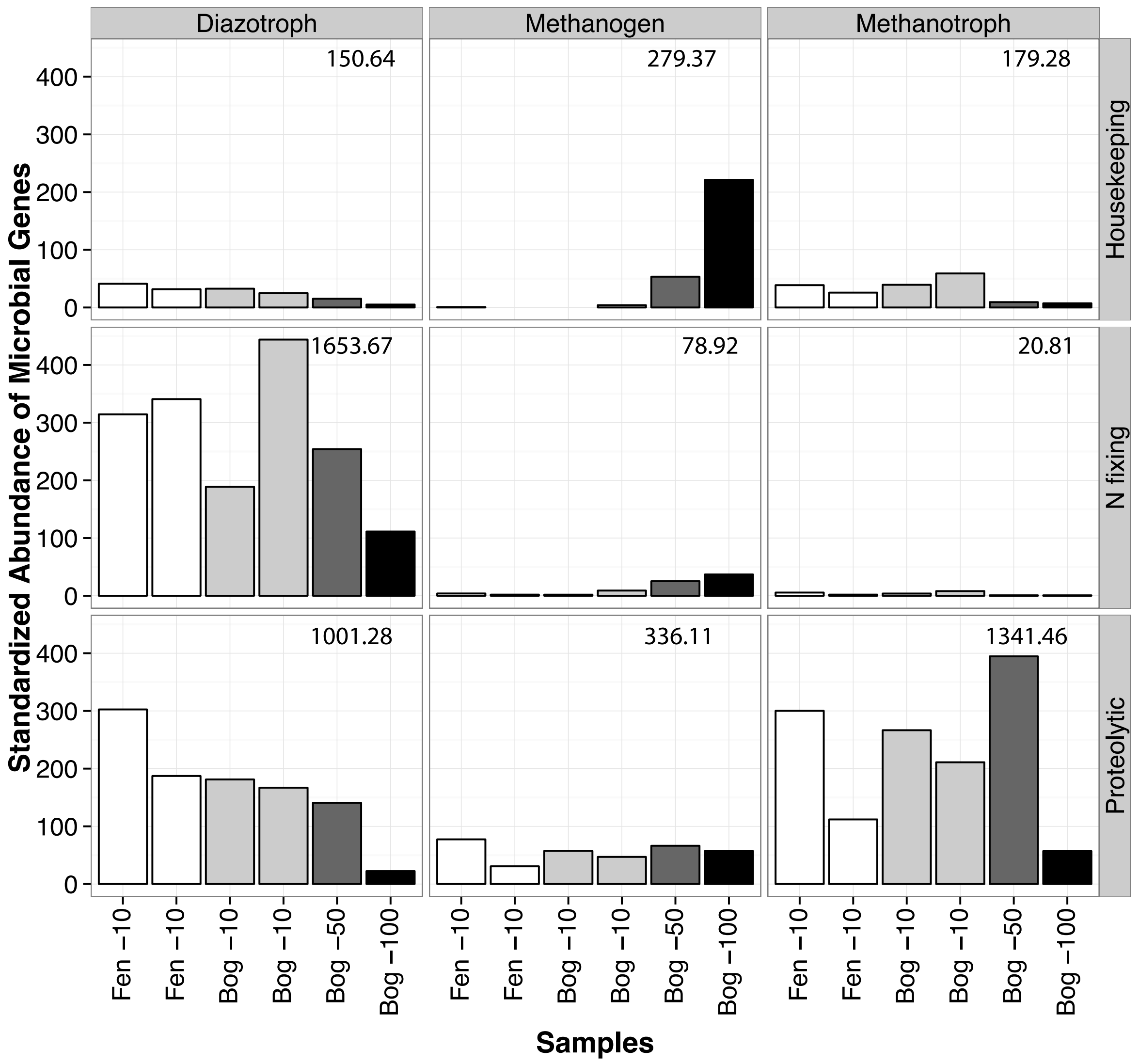

Figure S1. The abundance of genes attributed to diazotrophy, methanogenesis, and methanotrophy in housekeeping, nifH, and all protease genes. The number in each panel represents the total standardized abundance of all gene assemblies identified for the particular microbial group.

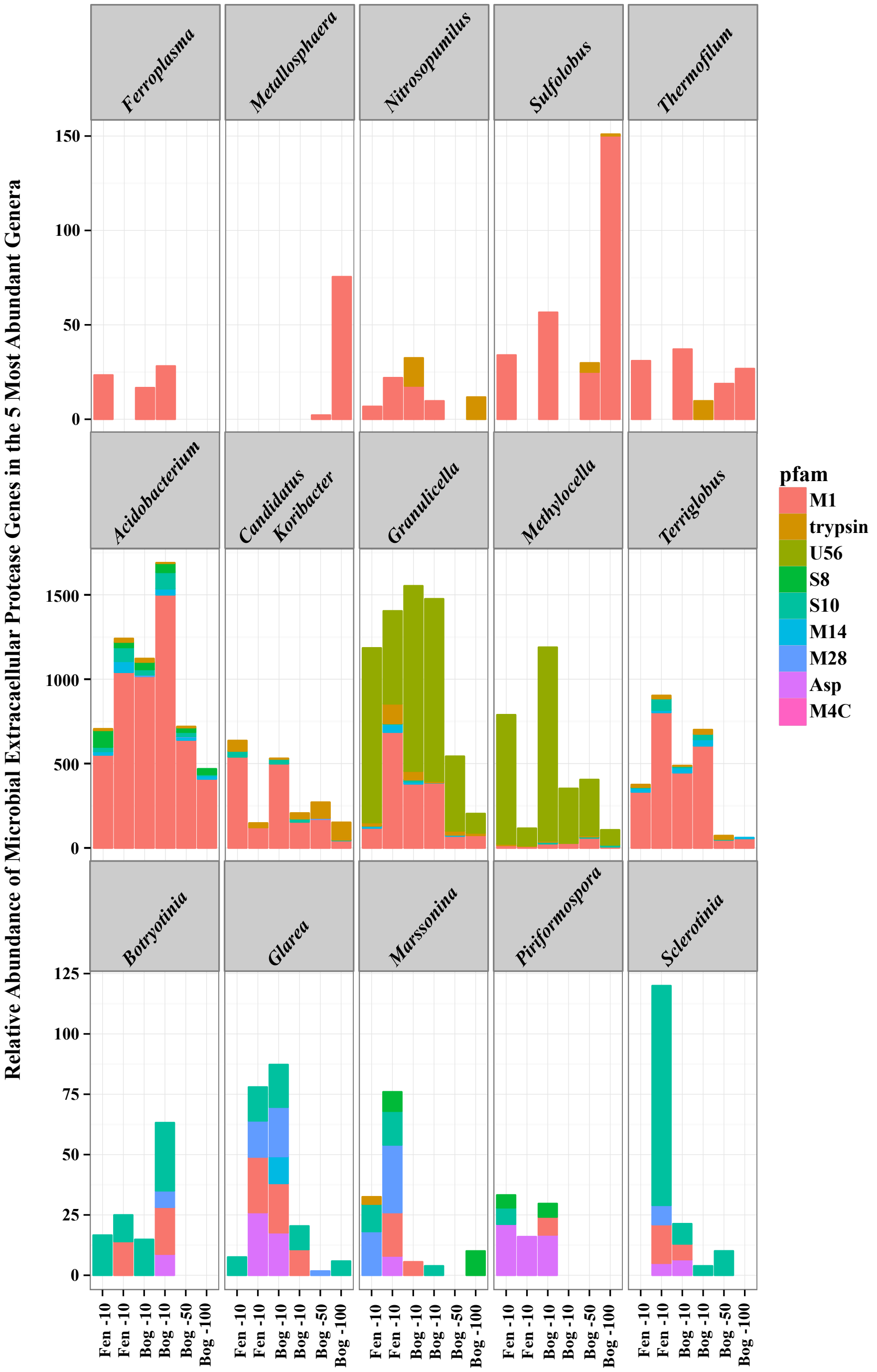

Figure S2. The abundance of protease genes attributed to the five most abundant genera within each kingdom. The top, middle, and bottom rows show archaea, bacteria, and fungi respectively.
